# Another Georgia Wonder Woman

**Published:** 1888-04

**Authors:** 


### ANOTHER GEORGIA WONDER WOMAN.

### SHE DOES MORE ASTOUNDING TRICKS THAN DID LULA HURST.

Very little idea of the mystery which surrounds Mrs. Dixie Jarrett Haygood, who will soon start out on a tour over the world, can be obtained until she is seen in her marvellous performances. In electric tricks she does even more than Miss Hurst. For instance, a person is blindfolded. An article is hidden, and then she places her hand lightly on the shoulder of the blindfolded person, who goes, without knowing why, directly to the hidden article. Very recently this feature of her performance was given a severe test. A pin was driven into the wall as high as the hand could reach. A lady had been blindfolded, and was to find what was hidden and the locality. The instant Mrs. Haygood's hand was placed upon the lady's shoulder she walked direct to the place and took the pin from the wall.

The slate writing of Slade is feeble when compared with that of Mrs. Haygood. A small pencil is laid upon a slate and the slate is then placed where, seemingly, writing could not be done; under a wardrobe, for instance. Answers to questions were made, and each time the answer was satisfactory to the asker. She has received hundreds of dollars in money and valuables, by being thus able to obtain from somewhere proper answers to questions.

But she is averse to this feature, and will not show it on every occasion. She does not know where the power comes from, and offers no explanation.

In her early days, when but a child, and before she had learned to write, she could cause messages to be written on slates. Among the many instances is this: Whenever the slate would be written on she would become frightened, and was of the opinion that it was done by some other person. One day she decided to test it herself. She thought of a verse in the Bible, "God is Love," and placed the slate where she knew it could not be touched. When a sufficient time had elapsed she examined the slate, and the words "God is Love" were written there in large letters.

An Episcopal minister doubted her ability to do such things, and resolved to put her to a test. He wrote a question on a piece of paper, tore off a piece, and rolling up the fragment upon which the question was written, placed and kept it in his mouth. The other portion of the paper was placed upon a table and Mrs. Haygood was called upon to give a reply. This was done. The answer was correct, the two pieces of paper were compared, and the minister was so confounded with the fact that he left the house at once.—*Savannah News.*

